# Spie charts for quantifying treatment effectiveness and safety in multiple outcome network meta-analysis: a proof-of-concept study

**DOI:** 10.1186/s12874-020-01128-2

**Published:** 2020-10-28

**Authors:** Caitlin H. Daly, Lawrence Mbuagbaw, Lehana Thabane, Sharon E. Straus, Jemila S. Hamid

**Affiliations:** 1 Department of Health Research Methods, Evidence, and Impact, McMaster University, McMaster University Medical Centre, 1280 Main Street West, 2C Area, Hamilton, Ontario L8S 4K1 Canada; 2 Population Health Sciences, Bristol Medical School, University of Bristol, Canynge Hall, 39 Whatley Road, Bristol, BS8 2PS UK; 3 Biostatistics Unit, Father Sean O’Sullivan Research Centre, St Joseph’s Healthcare Hamilton, 50 Charlton Avenue East, Hamilton, Ontario L8N 4A6 Canada; 4 Knowledge Translation Program, Li Ka Shing Knowledge Institute, St. Michael’s Hospital, 209 Victoria Street, Toronto, Ontario M5B 1TB Canada; 5 Department of Medicine, Faculty of Medicine, University of Toronto, C. David Naylor Building, 6 Queen’s Park Crescent West, Third Floor, Toronto, Ontario M5S 3H2 Canada; 6 Department of Mathematics and Statistics, STEM Complex, University of Ottawa, room 336, 150 Louis-Pasteur Private, Ottawa, Ontario K1N 6N5 Canada

**Keywords:** Network meta-analysis, Ranking, SUCRA, Spie chart, Radar plot, Multiple outcomes

## Abstract

**Background:**

Network meta-analysis (NMA) simultaneously synthesises direct and indirect evidence on the relative efficacy and safety of at least three treatments. A decision maker may use the coherent results of an NMA to determine which treatment is best for a given outcome. However, this evidence must be balanced across multiple outcomes. This study aims to provide a framework that permits the objective integration of the comparative effectiveness and safety of treatments across multiple outcomes.

**Methods:**

In the proposed framework, measures of each treatment’s performance are plotted on its own pie chart, superimposed on another pie chart representing the performance of a hypothetical treatment that is the best across all outcomes. This creates a spie chart for each treatment, where the coverage area represents the probability a treatment ranks best overall. The angles of each sector may be adjusted to reflect the importance of each outcome to a decision maker. The framework is illustrated using two published NMA datasets comparing dietary oils and fats and psoriasis treatments. Outcome measures are plotted in terms of the surface under the cumulative ranking curve. The use of the spie chart was contrasted with that of the radar plot.

**Results:**

In the NMA comparing the effects of dietary oils and fats on four lipid biomarkers, the ease of incorporating the lipids’ relative importance on spie charts was demonstrated using coefficients from a published risk prediction model on coronary heart disease. Radar plots produced two sets of areas based on the ordering of the lipids on the axes, while the spie chart only produced one set. In the NMA comparing psoriasis treatments, the areas inside spie charts containing both efficacy and safety outcomes masked critical information on the treatments’ comparative safety. Plotting the areas inside spie charts of the efficacy outcomes against measures of the safety outcome facilitated simultaneous comparisons of the treatments’ benefits and harms.

**Conclusions:**

The spie chart is more optimal than a radar plot for integrating the comparative effectiveness or safety of a treatment across multiple outcomes. Formal validation in the decision-making context, along with statistical comparisons with other recent approaches are required.

## Background

Health technology assessments and clinical guidelines are increasingly being supported by evidence synthesised through network meta-analysis (NMA) [1, 2]. The main output from an NMA is a coherent set of relative treatment effects, based on pooled direct and indirect evidence typically contributed by randomised controlled trials (RCTs) [3, 4]. The estimated treatment effects relative to a common comparator may then be used to inform a ranked list of treatments, from which knowledge users may be able to deduce which treatment is best for a given clinical problem.

Interpreting NMA results is challenging, particularly as the number of treatments and outcomes increase. Several pieces of literature have aimed to ease the interpretative burden of NMA. For example, three graphical tools were developed to display key features of an NMA (i.e. relative effects and their uncertainty, probabilities of ranking best, and between-study heterogeneity) for a single outcome [5]. The rank heat plot has been proposed as a visual tool for presenting NMA results across multiple outcomes [6]. However, knowledge users could also benefit from the quantification of the overall integrated results across multiple outcomes to facilitate interpretation in a more objective way. This is particularly important in situations where the comparative rankings of treatments on each outcome contradict each other.

Radar plots are often used as a visualisation tool to communicate multivariate data [7]. Recently, they have been used to visually compare the surface under the cumulative ranking curves (SUCRAs) in an NMA evaluating multiple interventions for relapsing multiple sclerosis [8]. Another NMA on dual bronchodilation therapy for chronic obstructive pulmonary disease has compared the area within radar charts of SUCRA values to deliver a single ranking of their efficacy-safety profile [9]. However, in this NMA, the quantification of the area weighed each outcome equally, which may not reflect a knowledge user’s preferences. The use of radar plots for the purpose of comparing the overall performance of treatments is also limited by the fact that the area depends on the ordering of the outcomes on the plot. For this reason, the spie chart has been suggested as a better alternative [10].

A spie chart is a combination of two pie charts, where one is superimposed on another, allowing comparisons between two groups on multiple attributes [11]. For example, in the context of NMA, this could be the comparison of a treatment against a hypothetical treatment that is uniformly the best across multiple outcomes. The former’s area will be a fraction of the latter’s, thereby facilitating the comparison of multiple treatments in a manner similar to comparing areas on a radar chart.

To address the aforementioned gaps and limitations, the objective of this paper is to lay the groundwork for conceptualising a treatment’s likelihood of being the best overall in terms of its coverage area inside a spie chart. This circular plot may be divided into segments representing a treatment’s level of efficacy or safety for each outcome. We provide a methodological framework and assess the feasibility of adapting the area on a spie chart to numerically integrate the efficacy and safety of treatments estimated by NMAs of multiple outcomes. Since radar plots have not been formally investigated and generalised for NMA, we also present the area on a radar plot and compare it to that of spie charts. We illustrate how the spie chart may be used to overcome the limitations of the radar plot, as well as its flexibility for further adaptations.

## Methods

### Measuring the coverage area inside a spie chart

**Fig. 1:**
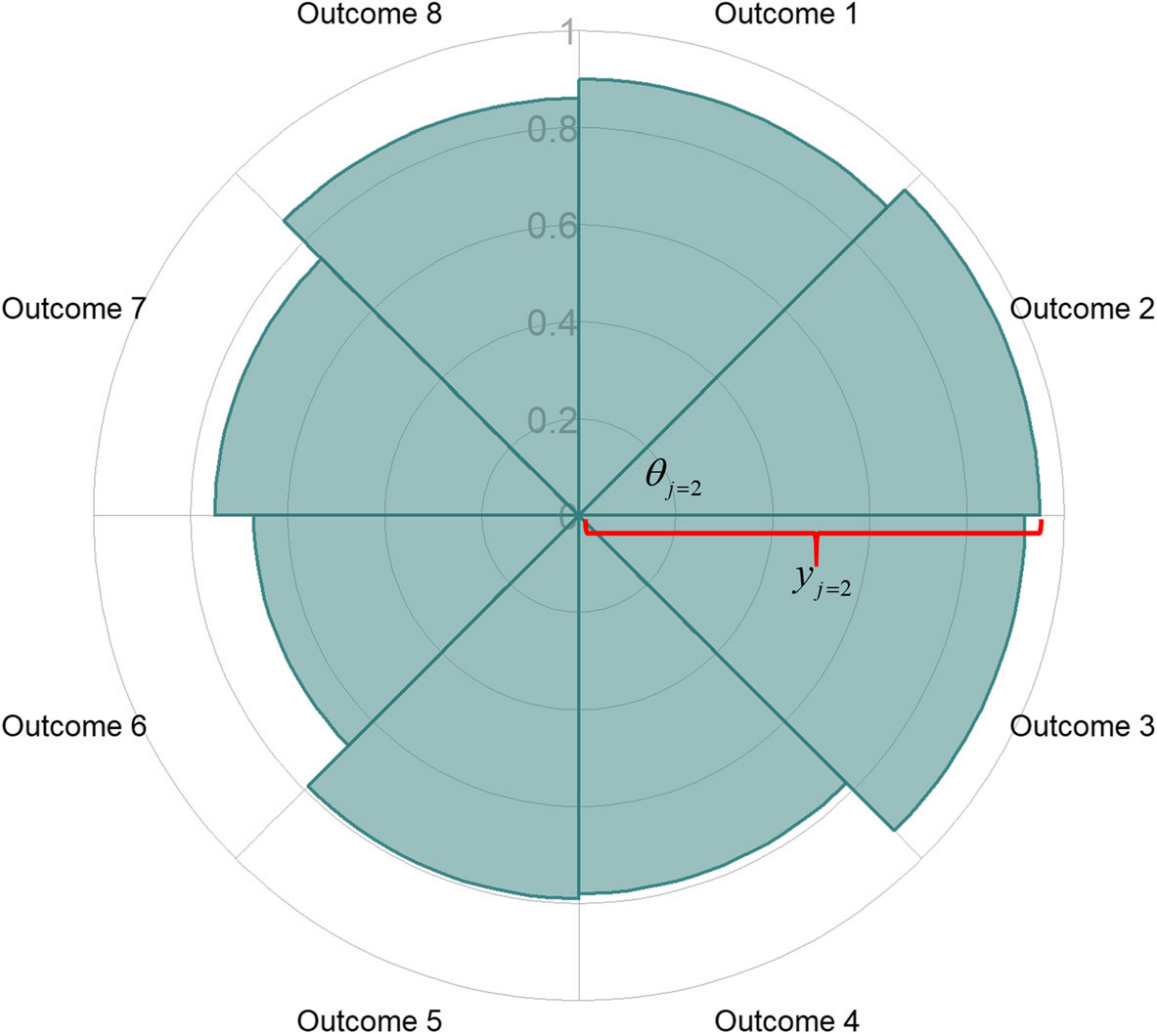
Example spie chart informed by the values of 8 outcomes. To calculate the area of sector *j* = 2, the required parameters are denoted: *θ*_*j* = 2_ is a known angle, *y*_*j* = 2_ is the radius of sector *j* = 2, equal to the value of outcome 2

Consider for example a situation where the performance of a treatment has been measured in terms of *J* = 8 outcomes valued between 0 and 1. Simulated values are plotted on a spie chart in Fig. 1. In general, the resulting shape on any spie chart is a series of *J* sectors, each with their own radius equal to the value of the *J* outcome measures. The area covered by these sectors may be calculated as the sum of the areas of the individual sectors.

In Fig. 1, the shaded area, *A*, is the sum of the area of the 8 sectors, *A*_*j*_, *j* = 1, ..., 8

$$ {A}_j=\frac{1}{2}{\theta}_j{y}_j^2, $$

where *y*_*j*_ and *θ*_*j*_ are the radius and angle of sector *j*, respectively. In Fig. 1, all angles are equal, i.e., ${\theta}_j=\frac{2\pi }{8}=\frac{\pi }{4}$ radians, and the shaded area on the spie chart is then:

$$ A=\frac{\pi }{8}\sum \limits_{j=1}^8{y}_j^2, $$

which is an average of the squared values of the 8 outcomes, scaled by a factor of *π*. In general, the shaded area within a spie chart informed by *J* ≥ 1 outcomes for an intervention is

$$ A=\frac{1}{2}\sum \limits_{j=1}^J{\theta}_j{y}_j^2. $$

### Choice of outcome measure

To enable a fair comparison of the areas defined by the treatments’ performances across multiple outcomes, the outcomes should be plotted on the same or comparative scales. This is not the case in most NMA studies involving multiple outcomes. As such, ranking probabilities and their summaries (e.g. the Surface Under the Cumulative RAnking curve (SUCRA) or P-score) may provide valid measures for this purpose [12, 13]. These measures transfer the comparative relative effects to a scale between 0 and 1. Alternatively, the posterior mean or median ranks may be used. However, note that the probability of a treatment ranking best should be avoided because treatments with high uncertainty around their estimated effects are likely to be ranked best [14], and this ranking probability has the potential to be biased [15]. SUCRA values, which are calculated in a Bayesian framework, provide a less sensitive and less biased alternative to rank treatments. The posterior mean rank is a scaled transformation of SUCRA, and the P-score is its frequentist equivalent [13]. These measures account for the uncertainty of a treatment’s relative effect, and are thus preferable [12].

Another option may be to use the absolute probabilities of response or risk for each treatment, as was done in a multicriteria decision analysis of statins [16]. Note that NMA pools relative effects such as log-odds ratios. To obtain estimates of the absolute probabilities for all treatments, an estimate of the absolute effect (e.g., log-odds) of a treatment in a contemporary population of interest must be assumed. This may be any treatment in the network [2]. The assumed absolute effect of this treatment would then be applied to the relative effects (e.g., log-odds ratio) vs. the chosen treatment, to obtain estimates of absolute effects (e.g., log-odds) of all other treatments, which may be subsequently converted into probabilities [2, 17]. If an NMA pools evidence on important outcomes measured on a continuous scale, response rates may be estimated [18] or standardised mean differences may be converted to log-odds ratios [19], provided that the underlying assumptions of these conversions are reasonable for the data. Note that plotting absolute probabilities of response or risk would limit the generalisability of the area to the population informing the assumed absolute effect of the chosen reference treatment.

In this paper, to simplify the presentation of our novel methodological framework, we use SUCRA values as a measure of the comparative effectiveness and safety of the treatments. We would like to highlight that this choice is made without loss of generality and the method is valid for any other measure.

### Standardised area inside a spie chart

To facilitate interpretation of the coverage area inside a spie chart, we standardise it by the maximum possible area. Its interpretation would then be comparable to the interpretation of SUCRA [12]. As such, the minimum possible standardised area of 0 corresponds to a treatment that always ranked the worst (i.e., SUCRA = 0 across all outcomes). The maximum possible standardised area of 1 corresponds to a treatment that always ranked best for each outcome (i.e., SUCRA = 1 across all outcomes).

First, consider the maximum possible area of each sector in a spie chart defined by SUCRA,

$$ {A_j}^{\mathrm{max}}=\frac{1}{2}{\theta}_j{(1)}^2=\frac{\theta_j}{2}. $$

If there are *J* outcomes and the angles of each sector are equal, i.e., ${\theta}_j=\frac{2\pi }{J},j=1,...,J$, then the maximum possible area on a spie chart is

$$ {A}^{\mathrm{max}}=\frac{1}{2}\sum \limits_{j=1}^J\left(\frac{2\pi }{J}\right)=\frac{J}{2}\left(\frac{2\pi }{J}\right)=\pi . $$

In fact, regardless of the size of the individual sector’s angles *θ*_*j*_, as long as the outcome measure *y*_*j*_ can range from 0 to 1, the spie chart consists of a unit circle. Consequently, *A*^max^ = *π*, for all 0 ≤ *θ*_*j*_ ≤ 2*π*. Therefore, in general, the standardised area on a spie chart consisting of outcome measures ranging between 0 and 1 is

$$ {A}^{std}=\frac{1}{2\pi}\sum \limits_{j=1}^J{\theta}_j{y}_j^2 $$

where *y*_*j*_ and *θ*_*j*_ are the outcome measure (e.g., SUCRA value) and angle of the sector corresponding to outcome *j*, respectively. Note that 0 ≤ *θ*_*j*_ ≤ 2*π*, where *θ*_*j*_ = 0 implies outcome *j* does not contribute the area, and *θ*_*j*_ = 2*π* implies outcome *j* is the sole contributor to the area. In the case of equal angles, the standardised area on a spie chart for a given treatment is a weighted average of the squared outcome measures,

$$ {A}^{std}=\frac{1}{J}\sum \limits_{j=1}^J{y}_j^2, $$

provided that the outcomes are measured on a scale between 0 and 1.

### Incorporating stakeholder preferences

An advantage of the spie chart’s circular design is the ability to incorporate preferences of the knowledge user. Some outcomes may be more important than others, and this can be incorporated in the plots by adjusting the contribution each outcome has to the overall area. By adjusting the angles of the sectors in a spie chart, we can adjust the proportion of the chart each sector covers. Noting that the sum of the angles in a spie chart must be 2*π*, given a set of weights, *w*_*j*_, *j* = 1, ..., *J* for a set of *J* outcomes, the corresponding angles may be calculated as

$$ {\theta}_j=\frac{2\pi {w}_j}{\sum \limits_{j=1}^J{w}_j}. $$

There are various ways to derive the contribution of the outcomes in terms of weights, which may be informed by preferences supported by evidence in the literature or through weights elicited from knowledge users themselves. For example, risk prediction or prognostic models may be used to inform the weights of outcomes when the goal is to reduce the risk of an unfavourable event or disease such as cardiovascular disease (CVD). If all outcomes are included in a regression model, and measured in the same units, the magnitude of the unstandardised coefficients, *β*_*j*_, *j* = 1, ..., *J*, capture the influence each outcome has on the overall risk, adjusted for any additional factors included in the model:

$$ {w}_j=\frac{\mid {\beta}_j\mid }{\sum \limits_{j=1}^J\mid {\beta}_j\mid }. $$

If the outcomes are measured on different scales, then standardised coefficients may be considered. There are more optimal approaches to deriving the relative importance of predictors (e.g., outcomes) when individual patient data (IPD) are available to create multiple regression models [20]. In fact, the use of standardised coefficients for this purpose has been criticised because the dependencies between predictors are not fully taken into account [21]. Nevertheless, researchers undertaking NMA often have limited resources in terms of time and access to IPD, and thus have to make secondary use of aggregate or summary level data.

If there are important dependencies between the outcomes, this should be accounted for at the synthesis stage. There are several approaches available for this and guidance is provided by López-López and colleagues [22] and multi-parameter evidence synthesis methods should also be considered [2]. Nevertheless, if there is a need to avoid double counting the contribution of related outcomes, and we know the correlations between them, the contribution of each outcome to the overall area can be adjusted. The weights of each outcome may be informed by a *J* × *J* correlation matrix, denoted as

$$ {\displaystyle \begin{array}{ccccc}1& {\rho}_{12}& {\rho}_{13}& \cdots & {\rho}_{1J}\\ {}{\rho}_{21}& 1& {\rho}_{23}& \cdots & {\rho}_{2J}\\ {}{\rho}_{31}& {\rho}_{32}& 1& \cdots & {\rho}_{3J}\\ {}\vdots & \vdots & \vdots & \ddots & \vdots \\ {}{\rho}_{J1}& {\rho}_{J2}& {\rho}_{J3}& \cdots & 1\end{array}} $$

However, since correlation can be negative, the squares of the pairwise correlations, i.e., the coefficients of determination, i.e., ${R}_{ij}^2={\rho}_{ij}^2$ should be used instead. The weight of each outcome can then be proportional or equal to the marginal sums of the squared correlation matrix:

$$ {w}_j=\sum \limits_{i=1}^J{\rho}_{ij}^2,i=1,...,J. $$

Finally, there are several methods for eliciting preferences from decision makers, such as direct rating, where the decision makers rate outcomes on a scale from 1 to 100 and weights are derived by normalising these scores [23]. Regardless of the method to inform the weights, the application of the proposed framework remains the same.

### Selecting outcomes to inform the area

The number of outcomes that may be plotted on a spie chart ranges from one to infinity. Nevertheless, a knowledge user would not benefit from either extreme. The purpose of the spie chart is to facilitate the combination of multiple outcomes, accounting for the desired contribution of the overall summary. As such, a minimum of two outcomes is sensible for this purpose. Plotting an overwhelming number of outcomes will not be visually appealing, although the area inside a spie chart is intended to overcome the visual interpretative burden. Increasing the number of outcomes will limit the contribution of important outcomes, to a degree that depends on the weights. Researchers presenting results of an NMA should be wary of this, though they do not need to restrict themselves to a maximum number of outcomes.

Outcomes which are critical to the decision-making process should be plotted on the spie chart. For example, any outcomes for which lack of evidence would exclude a treatment from consideration should be plotted. Evidence for any plotted outcome should be available for every treatment under consideration. It is important that every treatment is compared based on the same set of outcomes. If evidence on a critical outcome is not available for a treatment within a decision set, then imputation methods may be considered at the NMA stage [24].

Efficacy and safety outcomes should be plotted on two separate spie charts for each treatment, as it is important for a knowledge user to recognise that a very effective treatment may not be safe. Plotting these on the same spie chart and summarising the area inside as a single numerical value may mask important information on harms. A knowledge user should be able to simultaneously compare both the benefits and the harms of a treatment. This is possible by plotting the area inside an efficacy spie chart against the area inside a safety spie chart on a scatter plot [25]. Points towards the upper right quadrant of a scatter plot (e.g., towards (1,1)) would represent the most efficacious and safe treatment.

### Summary of steps

To summarise, our proposed method can be organised into the following steps:

1. Determine the final list of treatments to be compared and for which outcomes. All treatments should have evidence on the outcomes plotted on the spie charts.
2. Determine the outcome measure. Outcomes should be plotted in the same units and non-negative measures are recommended. If this is a ranking measure (e.g., probability of ranking best, P-score, or SUCRA), then separately calculate the ranking probabilities based on the subset of treatments determined in Step 1.
3. Determine the weights, *w*_*j*_ of each outcome *j* = 1, ..., *J* and the corresponding angle ${\theta}_j=\frac{2\pi {w}_j}{\sum \limits_{j=1}^J{w}_j}$ .
4. Plot the efficacy and safety spie charts for each treatment. One option is to make use of the ggplot2 package in the free and open source R statistical software [26, 27], using the R code provided in Additional File 1.
5. Calculate and compare the area inside the spie charts. The standardised area inside a spie chart consisting of outcomes measured on a scale between 0 and 1 is

$$ {A}^{std}=\frac{1}{2\pi}\sum \limits_{j=1}^J{\theta}_j{y}_j^2 $$

where *y*_*j*_ is the measure of outcome *j*, *j* = 1, ..., *J*, plotted on the spie chart. Any calculator or software may be used to apply this formula.

6. Plot the area inside the efficacy spie charts against the area inside the safety spie charts on a scatter plot.

## Results

In this section, our proposed framework is illustrated using results from two published reviews [28, 29]. At the same time, we empirically compare the use of the spie chart and the radar plot for quantifying a treatment’s overall performance. The formula for the standardised area inside a radar plot has been derived in Additional File 2. The two reviews used in this section were selected as all interventions have complete outcome information, and they highlight conceptual issues that drive the development of this framework. The first example illustrates one way of weighting outcomes of unequal importance to reflect the preferences of decision makers. Since there are four outcomes in this example, there are different ways to order the outcomes on a radar plot, and we show how this impacts the area inside a radar chart. In the second example, there are three outcomes, and thus one unique ordering of the outcomes which allows us to fairly compare the areas inside the radar plot and spie chart. The second example also underlines the importance of considering efficacy and safety outcomes separately. All analyses were performed using R [27]. We emphasize here that any observations made in these examples are for illustrative purposes only and should not impact clinical practice.

### Lipids study

The effects of thirteen dietary oils and fats on total cholesterol (TC), low-density lipoprotein cholesterol (LDL-c), high-density lipoprotein cholesterol (HDL-c) cholesterol, and triglycerides (TG), were investigated by Schwingshackl and colleagues [28]. Blood tests measuring these lipoproteins are carried out to assess a person’s risk for cardiovascular disease (CVD) [30]. The NMAs in this review pooled data from RCTs on thirteen treatments for the four outcomes of interest, and the SUCRA values are listed in Table 1. There is no treatment that clearly ranks the best across all outcomes.

**Table 1:** SUCRA values and rankings produced based on all trials included in [28]

Treatment	Outcome							
	Total Cholesterol^a^		LDL-c^a^		HDL-c^b^		Triglycerides^a^	
	SUCRA	Rank	SUCRA	Rank	SUCRA	Rank	SUCRA	Rank
Safflower oil	0.90	1	0.82	1	0.06	13	0.68	3
Rapeseed	0.85	2	0.76	2	0.53	7	0.58	7
Sunflower oil	0.72	4	0.71	4	0.57	5	0.61	6
Corn oil	0.72	4	0.66	6	0.29	11	0.66	4
Hempseed oil	0.61	5	0.69	5	0.59	4	0.63	5
Soybean oil	0.59	7	0.50	8	0.13	12	0.72	2
Flaxseed oil	0.59	7	0.71	4	0.47	9	0.56	8
Olive oil	0.43	8	0.37	9	0.52	8	0.32	10
Beef fat	0.41	9	0.50	8	0.74	3	0.06	13
Palm oil	0.34	10	0.33	11	0.80	2	0.74	1
Coconut oil	0.22	11	0.33	11	0.88	1	0.29	11
Lard	0.11	12	0.10	12	0.55	6	0.50	9
Butter	0.03	13	0.02	13	0.37	10	0.17	12

^a^Higher values of SUCRA reflect treatments that are better in terms of reducing levels of these lipids

^b^Higher values of SUCRA reflect treatments that are better in terms of increasing levels of this lipid

Note that, lower values of TC, LDL-c, and triglycerides are preferred, while higher values of HDL-c are preferred. The SUCRA values were computed in this NMA so that higher values of SUCRA reflect the preferred direction (i.e., improvement) of the outcomes. This is important when plotting outcomes on a spie chart, so that larger areas reflect treatments that are better at improving outcomes.

#### Spie chart

**Fig. 2:**
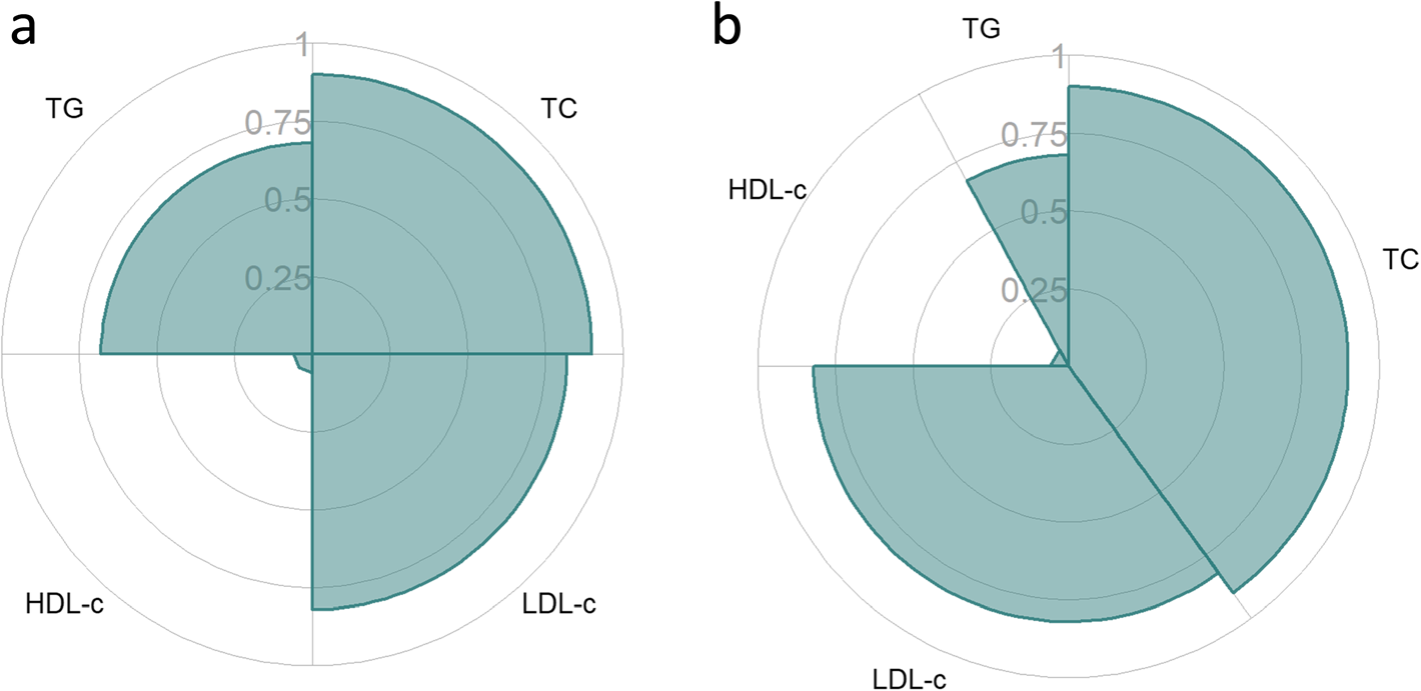
Two possible spie charts of the SUCRA values corresponding to Safflower oil in [28]. The plot in **a** weighs each outcome equally, since they have the same angles. The plot in **b** weighs the outcomes based on a coronary heart disease risk prediction model for women aged 50 - < 65 years [31]

To compare the rankings of areas inside a spie chart, we first calculated the standardised areas, assuming equal weights i.e., equal angles (Table 2). The area corresponding to the spie chart for Safflower oil is displayed in Fig. 2a. The percentages of the unit circle covered by the shaded areas for each treatment are small (Table 2), indicating that there is no treatment which is certainly the best across all outcomes. Knowing this, a stakeholder may then direct their attention to differences, if any, between treatments for more important outcomes.

**Table 2:** Standardised areas inside spie charts of SUCRA values from multiple outcomes

Treatment	Outcomes weighted equally		Outcomes weighted for women 50 - < 65 years^a^	
	Standardised Area	Rank	Standardised Area	Rank
Safflower oil	0.150	1	0.597	1
Rapeseed	0.147	2	0.566	2
Sunflower oil	0.132	3	0.469	3
Corn oil	0.113	5	0.409	4
Hempseed oil	0.123	4	0.406	5
Soybean oil	0.087	8	0.271	7
Flaxseed oil	0.107	7	0.378	6
Olive oil	0.053	11	0.176	11
Beef fat	0.075	10	0.248	8
Palm oil	0.109	6	0.237	9
Coconut oil	0.078	9	0.196	10
Lard	0.044	12	0.080	12
Butter	0.013	13	0.026	13

^a^ Outcomes are weighted differently according to a coronary heart disease risk prediction model for women aged 50 - < 65 years [31]

For illustrative purposes, we make use of a model built by Castelli et al., which built a multivariate regression model against coronary heart disease (CHD) [31]. This model was informed by data from the Framingham Study, and the reported regression coefficients for TC, LDL-c, HDL-c, and TG, for women aged 50 to less than 65 years old are 2.51, 2.19, − 1.05, 0.48, respectively. These coefficients are adjusted for systolic blood pressure, glucose, and cigarette smoking status. We can then calculate weights for each outcome, based on the absolute values of these coefficients. For TC,

$$ {w}_1=\frac{\mid 2.51\mid }{\mid 2.51\mid +\mid 2.19\mid +\mid -1.05\mid +\mid 0.48\mid }=\mathrm{0.40.} $$

The weights for LDL-c, HDL-c, and TG are 0.35, 0.17, and 0.08, respectively. The angle corresponding to TC may then be calculated as

$$ {\theta}_1=\frac{2\pi (0.40)}{1}=0.8\pi . $$

The angles for LDL-c, HDL-c, and TG are 0.7*π*, 0.34*π*, and 0.16*π*, respectively. The resulting area corresponding to the spie chart for Safflower oil is displayed in Fig. 2b. The standardised areas and ranks for each treatment, tailored to women aged 50 to less than 65 years are provided in Table 2. Based on this weighting scheme, the best treatment for reducing a 50 - < 65-year-old woman’s risk for CHD by improving lipid levels is Safflower oil.

#### Radar plot

In this example, there are four outcomes and thus four radii defining the radar plot. When using a radar plot, one must decide the order of the outcomes around the plot. The placement of the first outcome does not matter, but the ordering of the remaining *J* − 1 outcomes will impact the area enclosed in the radar plot [10]. There are $\frac{1}{2}\left(J-1\right)!$ options to order outcomes around a circle. The $\frac{1}{2}\left(4-1\right)!=3$ possible orderings of outcomes in this example are displayed in Supplementary Figure 2 in Additional File 3 for a single intervention, Safflower oil.

Summary of Findings tables in Cochrane Reviews may provide some guidance on how to order the outcomes, since the outcomes are listed in decreasing order of importance [32]. Of course, this importance ordering will vary across different stakeholders. For example, one Cochrane Review examining the effectiveness of a Mediterranean-style diet in preventing CVD has listed the decreasing order of importance of the four lipids as TC, LDL-c, HDL-c, TG [33]. Another Cochrane Review examining the effectiveness of polyunsaturated fatty acids in preventing CVD orders the importance of the lipids as TC, TG, HDL-c, LDL-c [34]. Nevertheless, these separate orderings will produce the same area, assuming the angles between the outcomes are equal, ${\theta}_j=\frac{2\pi }{4}=\frac{\pi }{2},j=1,2,3,4$. For example, the areas enclosed in the radar plots for Safflower oil (Supplementary Figure 2A&B, Additional File 3), based on the formula provided in Additional File 2, are

$$ {\displaystyle \begin{array}{rl}{A}_{\mathrm{Safflower},\mathrm{Rees}}^{std}& =\frac{1}{4}\left({y}_{TC}{y}_{LDL-c}+{y}_{LDL-c}{y}_{HDL-c}+{y}_{HDL-c}{y}_{TG}+{y}_{TG}{y}_{TC}\right)\\ {}& =\frac{1}{4}\left({y}_{TC}{y}_{TG}+{y}_{TG}{y}_{HDL-c}+{y}_{HDL-c}{y}_{LDL-c}+{y}_{LDL-c}{y}_{TC}\right)\\ {}& ={A}_{\mathrm{Safflower},\mathrm{Abdelhamid}}^{std}\end{array}} $$

There is only one other ordering that will produce a unique area: TC, HDL-c, LDL-c, TG. This is because of the triangles formed by TC & HDL-c and LDL-c & TG; these outcomes were not congruent in the plots generated by Rees’ and Abdelhamid’s orderings (Supplementary Figure 2C, Additional File 3). The standardised areas produced by these two ordered datasets, assuming equal angles, are provided in Table 3. The rankings of some of the treatments change, and although the differences between standardised areas may seem trivial in this example, this is an important feature of radar plots to highlight, as the differences could be exacerbated in other applications. For example, the areas for one treatment may be quite different if the outcomes are arranged in such a way that those reflecting higher scores alternate with those that have lower scores vs. an ordering where all outcomes with high scores are placed together.

**Table 3:** Standardised areas inside radar plots of SUCRA values from multiple outcomes

Treatment	Ordering A^a^		Ordering B^b^	
	Standardised Area	Rank	Standardised Area	Rank
Safflower oil	0.360	4	0.318	6
Rapeseed	0.462	1	0.447	1
Sunflower oil	0.426	2	0.422	2
Corn oil	0.333	6	0.328	5
Hempseed oil	0.396	3	0.397	3
Soybean oil	0.220	8	0.232	8
Flaxseed oil	0.337	5	0.335	4
Olive oil	0.164	10	0.168	10
Beef fat	0.161	11	0.182	9
Palm oil	0.305	7	0.258	7
Coconut oil	0.171	9	0.161	11
Lard	0.099	12	0.055	12
Butter	0.019	13	0.007	13

^a^Ordering A: TC, LDL-c, HDL-c, TG

^b^Ordering B: TC, TG, HDL-c, LDL-c

### Psoriasis example

The effectiveness and safety of seven biologic therapies plus placebo for treating psoriasis were investigated by Jabbar-Lopez and colleagues to support the development of a guideline [29]. Randomised trials informed the NMAs, which synthesised evidence on the following outcomes measuring efficacy: clear/nearly clear skin (defined as minimum residual activity, Psoriasis Area and Severity Index (PASI) > 90, or 0 or 1 on physician’s global assessment), mean change in dermatology life quality index (DLQI), and PASI 75 (defined as PASI > 75). The first 2 outcomes were deemed “critical” outcomes by the guideline development group, while the latter outcome, PASI 75, was deemed “important”. An additional outcome measuring safety, referred to in the review as tolerability or withdrawal due to adverse events, was also deemed an “important” outcome. For illustrative purposes, the published SUCRA values corresponding to each treatment under investigation are displayed in Table 4.

**Table 4:** SUCRA values and rankings produced based on all trials included in [29]

Treatment	Outcome							
	Clear/nearly clear		Mean change in DLQI		PASI 75		Withdrawal due to adverse events	
	SUCRA	Rank	SUCRA	Rank	SUCRA	Rank	SUCRA	Rank
Ixekizumab	0.99	1	0.70	3	0.96	1	0.14	7
Secukinumab	0.85	2	0.85	1	0.79	3	0.80	3
Infliximab	0.67	3	0.80	2	0.81	2	0.04	8
Ustekinumab	0.60	4	0.70	4	0.52	4	0.82	1
Adalimumab	0.46	5	0.51	5	0.49	5	0.81	2
Etanercept	0.28	6	0.31	6	0.28	6	0.46	6
Methotrexate	0.15	7	0.15	7	0.15	7	0.47	4
Placebo	0.00	8	0.00	8	0.00	8	0.47	5

As was the case in the lipids example, there is no treatment that is universally the best according to SUCRA across all outcomes. Ixekizumab has the largest SUCRA value in terms of the critical “Clear/nearly clear” outcome, but it is not the best in terms of the other critical outcome, mean change in DLQI. It also ranks the second worse in terms of tolerability, highlighting the importance of considering efficacy and safety outcomes separately.

#### Radar plot vs. spie chart

For illustrative purposes, we first combine the evidence on the three efficacy outcomes (clear/nearly clear, DLQI, PASI 75), considering them to be of equal importance (although the guideline committee suggested otherwise) [29] on both the spie chart and the radar plot. Since there are only three outcomes, there is only one way to arrange the order of the outcomes, and thus one unique area. As such, this example provides an opportunity to fairly compare the area on the radar plot with that on the spie chart.

The standardised areas on the radar plot and spie chart are provided in Table 5. The standardised areas for each treatment on both plots are quite similar, and the corresponding ranks are the same. Nevertheless, the efficacy outcomes equally contributed to the standardised area, which is unlikely to reflect a knowledge user’s preferences. There are some dependencies between the outcomes. For example, treatments that clear or nearly clear psoriasis for a large proportion of patients are also likely to have a higher proportion of patients that achieve a PASI score of at least 75. These dependencies should be accounted for using methods such as the ones suggested earlier in the Methods section.

**Table 5:** Standardised areas inside radar plots and spie charts of SUCRA values in [29]

Treatment	Radar Plot		Spie Chart	
	Standardised Area^a^	Rank	Standardised Area^a^	Rank
Ixekizumab	0.775	1	0.801	1
Secukinumab	0.686	2	0.687	2
Infliximab	0.572	3	0.578	3
Ustekinumab	0.362	4	0.370	4
Adalimumab	0.236	5	0.237	5
Etanercept	0.084	6	0.084	6
Methotrexate	0.022	7	0.022	7
Placebo	0.000	8	0.000	8

^a^These areas solely summarise the comparative ranking in terms of efficacy outcomes

#### Scatter plot of efficacy vs. safety

The purpose of this illustration is to show the consequences of naively plotting all efficacy and safety outcomes on a spie chart and summarising them with a single numerical value. As such, the standardised areas on a spie chart containing all efficacy and safety outcomes were calculated, assuming they were of equal importance. Of course, in practice, this is unlikely to be true. A knowledge user may want the contribution of the safety outcome to be the same as the contribution of the collection of efficacy outcomes. This is possible by dividing the spie chart in half, where the safety outcome is plotted on one half of the chart, and the three efficacy outcomes contribute equally to the other half. Nevertheless, the numerical summary of the coverage area will not allow a knowledge user to simultaneously compare the benefits and harms of the treatments, and so a scatter plot comparing the two is more desirable.

**Fig. 3:**
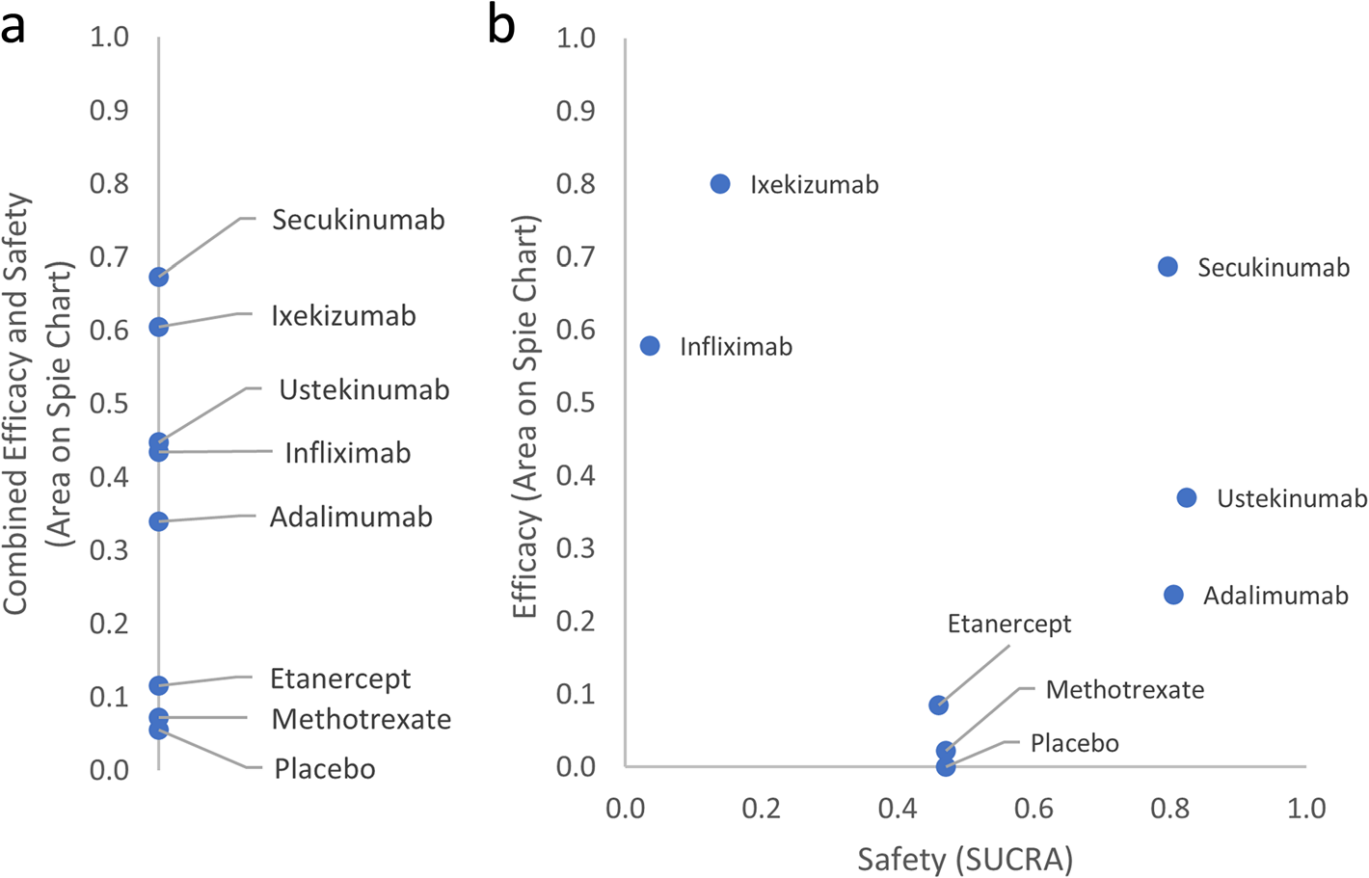
Comparison of two approaches for balancing efficacy and safety outcomes in [29]. In **a**, the areas inside a spie chart containing both efficacy and safety outcomes are plotted on a number line, where larger values (areas) are preferred. In **b** the areas inside a spie chart containing efficacy outcomes only are plotted against the SUCRA values for the single safety outcome on a scatter plot, where values in the top-right corner are preferred

The results show that Ixekizumab has the second highest SUCRA value (Fig. 3a), agreeing with the ranks solely based on efficacy (Table 5), but the message that it is one of the least tolerable is lost in this result (Table 4). The standardised area on the spie chart containing the efficacy outcomes only is plotted against the reported SUCRA values for the safety outcome in Fig. 3b. In this scatter plot, treatments in the top right corner are preferred. The benefit-risk trade-off is clearer for Ixekizumab, and Secukinumab seems to have the best benefit-risk ratio.

## Discussion

We have developed and presented a framework for obtaining the overall performance of treatments in NMA, summarised across all outcomes. Similar to SUCRA, the standardised area on a spie chart is a ratio of the maximum possible area, which a treatment could have if it always ranked best [12]. This paper lays the groundwork for integrating evidence across multiple outcomes, including some direction on how to incorporate key considerations for decision makers (e.g., outcome preferences). Table 6 presents a summary of graphical tools available for presenting multiple outcomes, where rank-o-grams [12, 36], standard scatter plots comparing two outcomes [35], the rank heat plot [6], radar plot, and spie chart are compared.

**Table 6:** Properties of graphical tools available to summarise multiple outcomes in NMA

Properties	Methodological Frameworks				
	Rank-o-grams	Scatter plot^a^	Rank heat plot	Radar plot	Spie charts
Summary type					
Numerical	✗	✗	✗	✓	✓
Does not depend on ordering of outcomes	–	–	–	✗	✓
Visual	✓	✓	✓	✓	✓
Single figure	✗	✗	✓	✗	✗
Outcome measures					
*Same units*					
Absolute effects	✗	✓	✓	✓	✓
P (best)	✓	✓	✓	✓	✓
P-score	✗	✓	✓	✓	✓
SUCRA	✗	✓	✓	✓	✓
All ranking probabilities	✓	✗	✗	✗	✗
Mean or median rank	✗	✓	✓	✓	✓
*Different units*					
Relative effects	✗	✓	✗	✗	✗
Outcome preferences					
Straightforward ability to weight outcomes	✗	✗	✗	✗	✓
Other considerations					
Can include > 2 outcomes	✓	✗	✓	✓	✓
Can accommodate missing outcomes	✓	✗	✓	✗	✗

^a^Scatter plots of two outcomes [35]

Radar plots have been used in the past to compare outcomes in health research. More recently, they have been used to summarise the performance of treatments in an NMA context [8, 9]. Despite this, there are several limitations of radar plots that reviewers should consider, and spie charts may be a more suitable alternative. A radar plot may be sufficient when evidence on three outcomes needs to be combined, and these three outcomes are of equal importance. If there are any additional outcomes, subjectivity can arise through the ordering of outcomes on the plot. Nevertheless, this may be mitigated by specifying outcome preferences a priori, which can be informed by preferences in Cochrane Summary of Findings tables or through a survey of stakeholders’ preferences.

Spie charts, however, are a more generalisable option and they have nicer mathematical properties compared to a radar chart. For example, the standardised area on a spie chart informed by a single outcome will output the same value that was inputted. In addition, adjusting the contribution of several outcomes on a spie chart is mathematically straightforward. Weighting schemes should be specified a priori to minimise subjectivity. This is also important when using coefficients from a risk prediction model to inform the weights, as it is important to select a risk prediction model that has been validated and covers the population of interest. Some risk prediction models may even present coefficients tailored to subgroups, as shown in the lipids example, permitting subgroup-specific ranks.

Nevertheless, the practice of using coefficients to inform the relative importance of predictors has been criticised [21, 37]. More optimal methods require individual patient data [20], which NMA researchers may not have access to. Formally eliciting the relative importance of outcomes from decision makers may offer a better alternative in the NMA context [23]. In the future, it would be useful to design a weighting scheme that accounts for both the dependencies between the outcomes, as well as the preferences of knowledge users.

This framework was illustrated using SUCRA values; however, other outcome measures could be used. Nevertheless, the cited examples of systematic reviews presenting evidence across outcomes through radar plots have done so using SUCRA values [8, 9]. Another recent review averaged SUCRA values on LDL-c, HDL-c, and TG to give an overall indication of the effectiveness of diets on the lipid profile [38]. SUCRA is an attractive measure to compare treatments across multiple outcomes as it summarises both the strength and uncertainty of the relative treatment effects on the same scale [13]. The standardised area inside a spie chart informed by SUCRA values clearly conveys the degree of uncertainty in the evidence across outcomes. This is because the outcome values are squared in the calculation of the area, and smaller SUCRA values, which indicate less plausibility or certainty in a treatment ranking best, are penalised. The standardised area for a particular treatment will only be close to 1 if there is large certainty supporting a treatment being more effective than all other treatments across all outcomes.

While a treatment may be very effective, it could also be unsafe, and so it is important to consider efficacy and safety outcomes separately and not summarise them with one measure. Efficacy and safety outcomes should be combined separately, and they may be simultaneously compared in scatter plots such as the one plotted in Fig. 3b for the psoriasis example. Nevertheless, we pause to reflect whether safety outcomes should be combined at all. A treatment’s harmful effects in terms of one outcome could be diluted by the appearance of its safety in terms of several other outcomes. It might be better to pool evidence on efficacy outcomes together as a single measure and then compare it against critical safety outcomes one by one.

Additional aspects of the evidence also need to be considered, such as the internal and external biases of the RCTs informing the networks. This goes beyond assessing whether the evidence supporting a treatment ranking best is at high risk of bias. The decision maker must grasp how the biased trials affect the network estimates, and this depends on the geometry of the networks and size of the trials. Sensitivity analyses which remove the trials at high risk of bias, threshold analyses, or CINeMA may provide some insight into this [39–43]. Methods for integrating such assessments into the spie chart should be explored in future work. For example, if CINeMA is used to evaluate the confidence in the NMA results [41–43], then an overall confidence rating for each outcome may be represented through colours or symbols on the spie chart for a given treatment.

There may be instances where there is no evidence on a treatment for a particular outcome. This treatment could still be included in the overall evaluation through spie charts, where a value of 0 is assumed. This would penalise the treatment’s performance for missing outcome data. However, if a treatment cannot be considered without information on a critical outcome, then perhaps it should be excluded from the evaluation of the overall performances. Note that SUCRA depends on the number of treatments informing it. As such, the number of treatments should be equal across all outcomes to allow fair comparison. If a treatment is excluded from the decision set, then it should not be included in the calculation of the ranking probabilities, and thus SUCRA.

## Conclusion

We have established the foundation of a framework that objectively summarises the comparative effectiveness of a treatment across multiple outcomes. This eliminates any subjectivity that may be introduced by a decision maker balancing contradictory rankings of treatments across different outcomes. The proposed framework is not meant to be a standalone presentation of the NMA results. Rather, it is intended to supplement the more detailed results that must be considered when evaluating the evidence. Forest plots or pairwise relative effect estimates should also be inspected to confirm whether there are any significant differences between treatments, a feature which may be masked by ranking statistics. Future research should investigate ways to adapt this framework when outcomes are missing for some treatments. The general approach should also be compared with existing numerical approaches for integrating ranks across outcomes [44, 45]. Moving forward, we recommend the spie chart over the radar plot for summarising effectiveness across multiple outcomes.

## Supplementary information

**Additional file 1:** R code for spie charts. R script containing function to generate and calculate area inside a spie chart.

**Additional file 2:** Measuring the area inside a radar plot. Describes the derivation of the standardised area inside a radar plot, as well as the difficulty in incorporating incorporate stakeholder preferences through the angles between the axes of a radar plot.

**Additional file 3: Supplementary Figure 2.** Three possible radar plots of the SUCRA values corresponding to Safflower oil in [26]. The plots in panel A and B have the same area, since they are the same shape flipped at the vertical axes. The plot in panel C has a different area due to the different triangles formed by TC & HDL-c and TC & LDL-c.

## Data Availability

All data generated or analysed during this study are included in this published article.

## Abbreviations

CHD: Coronary heart disease
CVD: Cardiovascular disease
DLQI: Dermatology life quality index
HDL-c: High-density lipoprotein cholesterol
IPD: Individual patient data
LDL-c: Low-density lipoprotein cholesterol
NMA: Network meta-analysis
PASI: Psoriasis area severity index
RCT: Randomised controlled trial
SUCRA: Surface under the cumulative ranking curve
TC: Total cholesterol
TG: Triglycerides
