# Supplementary material for: Spie charts for quantifying treatment effectiveness and safety in multiple outcome network meta-analysis: a proof-of-concept study
Source: BMC Med Res Methodol. 2020 Oct 28;20:266. doi: 10.1186/s12874-020-01128-2 (PMC7592566; doi:10.1186/s12874-020-01128-2)
Supplement: Supplementary file 1 — Additional file 1: R code for spie charts. R script containing function to generate and calculate area inside a spie chart. [file 12874_2020_1128_MOESM1_ESM.docx]

#####################################################################################

# Function to create a spie chart for a single treatment

# IMPORTANT NOTE: Calculation of area assumes outcomes are plotted on a scale between 0 and 1

#

# Uses ggplot2 package

#

# Requires the following input:

# outcome = vector of the outcome measures

# theta = vector of the calculated angles for each outcome (must add to 2*pi)

# outcome.range = the minimum and maximum possible values of the outcomes plotted

# outcome.label = vector of the outcome names

# yticksnum = number of equally spaced tick marks defining y-axis

#

#####################################################################################

library(ggplot2)

spie.chart <- function(outcome, theta, outcome.range=c(0,1), outcome.label, yticksnum=5){

spie.data <- data.frame(outcome = outcome, theta = theta)

pos <- 0.5 * (cumsum(theta) + cumsum(c(0, theta[-length(theta)])))

p <- ggplot(spie.data, aes(x = pos)) +

geom_vline(xintercept = cumsum(c(0, theta)), colour = "darkgrey", size = 0.2) +

geom_hline(yintercept = seq(min(outcome.range),max(outcome.range),length.out = yticksnum),

colour = "darkgrey", size = 0.2) +

geom_bar(aes(y = outcome), fill=rgb(0.2,0.5,0.5,0.5), width = theta, color = rgb(0.2,0.5,0.5,0.9),

stat = "identity") +

coord_polar(theta = "x") +

scale_x_continuous(labels = outcome.label, breaks = pos) +

scale_y_continuous(limits=c(min(outcome.range),max(outcome.range)),

breaks=seq(min(outcome.range),max(outcome.range),length.out = yticksnum)) +

theme(panel.grid.major.x = element_blank(), panel.grid.major.y = element_blank(),

panel.grid.minor.x = element_blank(), panel.grid.minor.y = element_blank(),

panel.background = element_blank(), axis.title = element_blank(),

axis.text.x=element_text(colour="black"),

axis.text.y = element_blank(), axis.ticks = element_blank(),

plot.margin = margin(0.5, 0, 0.5, 0, "cm")) +

# Add axis labels

annotate("text", x=0, y=seq(min(outcome.range),max(outcome.range),length.out = yticksnum),

label=round(seq(min(outcome.range),max(outcome.range),length.out = yticksnum),2),

colour="slategrey", hjust=1)

area <- (1/(2*pi*(max(outcome.range)-min(outcome.range))^2))*sum(theta*outcome^2)

return(list(p, paste("Area inside spie chart = ", round(area,2))))

}

# Sample spie chart

spie.chart(outcome=c(0.2,0.4,0.5,0.9), theta=c(0.1*pi,0.5*pi,0.7*pi,0.7*pi),

outcome.range=c(0,1), outcome.label = c("Outcome 1","Outcome 2","Outcome 3","Outcome 4"))
