# Supplementary material for: Spie charts for quantifying treatment effectiveness and safety in multiple outcome network meta-analysis: a proof-of-concept study
Source: BMC Med Res Methodol. 2020 Oct 28;20:266. doi: 10.1186/s12874-020-01128-2 (PMC7592566; doi:10.1186/s12874-020-01128-2)
Supplement: Supplementary file 2 — Additional file 2: Measuring the area inside a radar plot. Describes the derivation of the standardised area inside a radar plot, as well as the difficulty in incorporating incorporate stakeholder preferences through the angles between the axes of a radar plot. [file 12874_2020_1128_MOESM2_ESM.docx]

**Measuring the Area Inside a Radar Plot**

The traditional radar plot is equally divided by at least 3 axes or radii which measure an attribute of interest, e.g., health outcomes. To illustrate, Supplementary Figure 1 displays a radar plot that is informed by simulated values between 0 and 1 for $j=8$ outcomes.


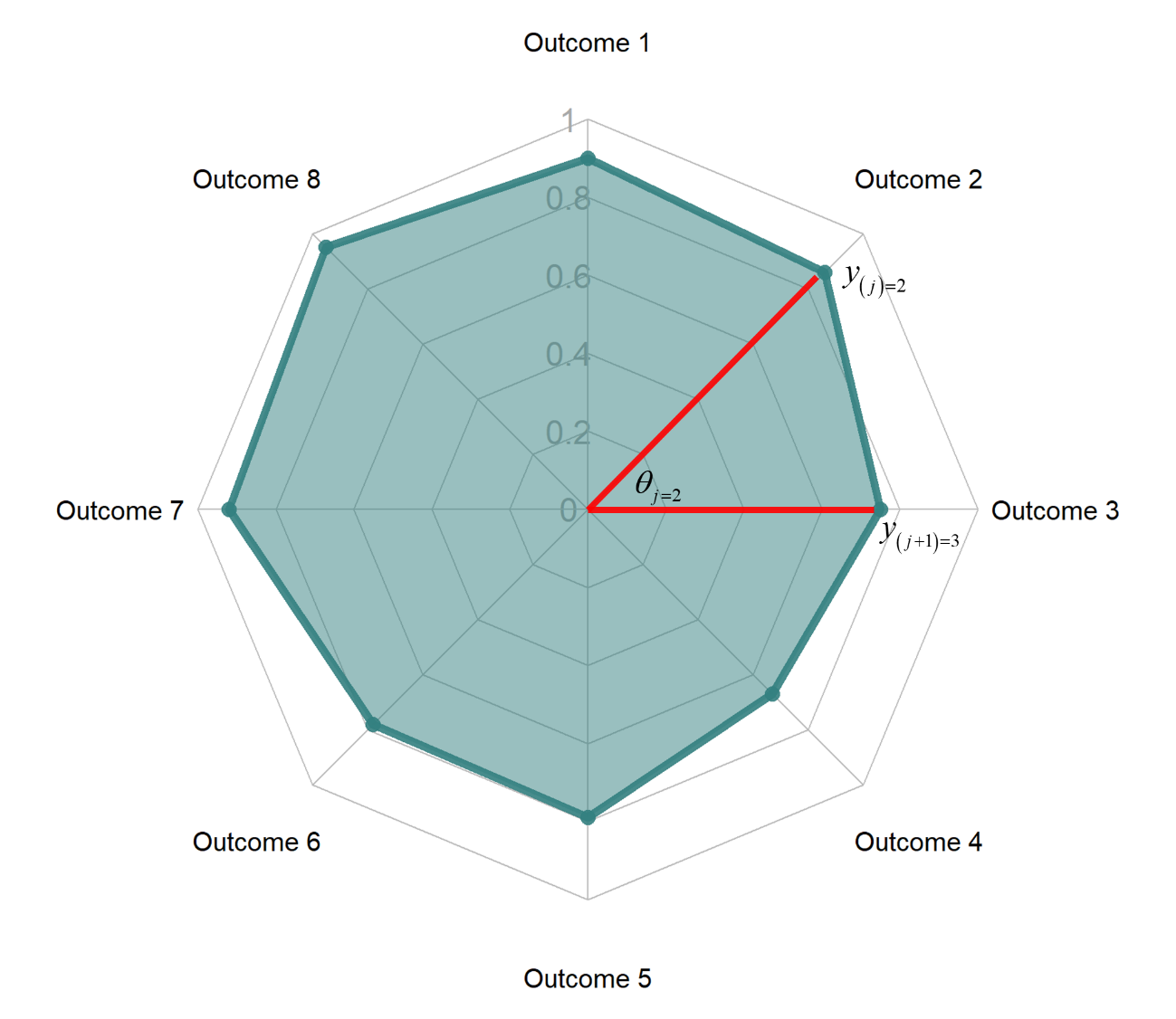


**Supplementary Figure 1:** Example radar plot informed by the values of 8 outcomes. To calculate the area of triangle $j=2$, the required parameters are denoted: $\theta_{j=2}$ is a known angle,$y_{\left(j\right)=2}$ and $y_{\left(j+1\right)=3}$ are the lengths of two sides of triangle $j=2$, which are equal to the values of outcomes 2 and 3, respectively.

The resulting shape on any radar plot is typically an irregular polygon, and the area it covers may be calculated as the sum of the areas of the triangles forming the shape. In Supplementary Figure 1, the area enclosed,$A$, is the sum of the area of 8 triangles,$A_{\Delta_{j}},j=1,...,8$,

$A_{\Delta_{j}}=\frac{1}{2}y_{\left(j\right)}y_{\left(j+1\right)}\sin\theta_{j}$,

where$y_{\left(j\right)},y_{\left(j+1\right)}$ are the lengths of the sides congruent to the angle of each triangle $j$, equal to the values of the outcome measure on the corresponding radii, and $\theta_{j}$ are the angles between the radii. Since the 8 radii defining this radar plot are equidistant, the angle corresponding to each triangle’s vertex at the centre are equal, i.e., $\theta_{j}=\frac{2\pi}{8}=\frac{\pi}{4}$ radians, and the area of inside this radar plot is then:

$A=\frac{1}{2}\sin\left(\frac{\pi}{4}\right)\sum_{j=1}^{8} y_{\left(j\right)}y_{\left(j+1\right)}$.

In general, the area within a radar plot informed by $J\geq3$ outcomes for an intervention is

$$A=\frac{1}{2}\sum_{j=1}^{J} y_{\left(j\right)}y_{\left(j+1\right)}\sin\theta_{j}^{*}$$

where $j=1,...,J$, $\left(j+1\right)=\left(1\right)$ when $j=J$, and $\theta_{j}^{*}=\theta_{j}$ if $0<\theta_{j}<\frac{\pi}{2}$ radians, otherwise$\theta_{j}^{*}$ is the related acute angle.

**Standardised Area on a Radar Plot**

The maximum possible area of each triangle in a radar chart is achieved when $y_{\left(j\right)}=y_{\left(j+1\right)}$= maximum possible value. When the maximum possible value is 1, as is the case for SUCRA, then

$A_{\Delta_{j}}^{\max}=\frac{1}{2}\left(1\right)\left(1\right)\sin\theta_{j}=\frac{1}{2}\sin\theta_{j}$.

If there are $J$ outcomes and the angles between the radii are equal, i.e., $\theta_{j}=\frac{2\pi}{J},j=1,...,J$, then

$A^{\max}=\frac{1}{2}\sum_{i=1}^{J} \sin\left(\frac{2\pi}{J}\right)=\frac{J}{2}\sin\left(\frac{2\pi}{J}\right)$.

So, in the case of equal angles, the standardised area on a radar chart for a given treatment is then

$$\begin{matrix} A^{std}=\frac{2}{J\sin\left(\frac{2\pi}{J}\right)}\frac{1}{2}\sin\left(\frac{2\pi}{J}\right)\sum_{j=1}^{J} y_{\left(j\right)}y_{\left(j+1\right)} \\ =\frac{1}{J}\sum_{j=1}^{J} y_{\left(j\right)}y_{\left(j+1\right)} \end{matrix}$$

where$y_{\left(j\right)},y_{\left(j+1\right)}$ are the SUCRA values congruent to the angle $\theta_{j}=\frac{2\pi}{J}$ of each triangle $j$. In general, the standardised area on a radar chart is

$$\begin{matrix} A^{std}=\frac{2}{\sum_{j=1}^{J} \sin\theta_{j}^{*}}\frac{1}{2}\sum_{j=1}^{J} y_{\left(j\right)}y_{\left(j+1\right)}\sin\theta_{j}^{*} \\ =\frac{1}{\sum_{j=1}^{J} \sin\theta_{j}^{*}}\sum_{j=1}^{J} y_{\left(j\right)}y_{\left(j+1\right)}\sin\theta_{j}^{*} \end{matrix}$$

where

$$\theta_{j}^{*}=\left\{\begin{matrix} \theta_{j} & \text{if }0<\theta_{j}\leq\frac{\pi}{2} \\ \pi-\theta_{j} & \text{if }\frac{\pi}{2}<\theta_{j}<\pi\end{matrix}\right.$$

and all other parameters are as defined above. Note that $0<\theta_{j}<\pi$ , since by definition, the sum of the angles inside a triangle is $\pi$ radians.

**Incorporating Stakeholder Preferences**

In the spie chart, we were able to incorporate a stakeholder’s preferences through the angles of the segments. However, this is not straightforward in a radar plot since the vertex angles are functions of 2 outcomes:

$\theta_{j}=\sin^{-1}\left(\frac{2A_{\triangle_{j}}}{y_{\left(j\right)}y_{\left(j+1\right)}}\right)$.

The spie chart is much more favourable plot for this purpose since the angle of a sector can be adjusted to solely adjust the contribution of 1 outcome:

$\theta_{j}=\frac{2A_{○_{j}}}{y_{j}^{2}}$ .

As such, we recommend spie charts when there is a need to weight each outcome differently.
