# Supplementary figures and images for: Spie charts for quantifying treatment effectiveness and safety in multiple outcome network meta-analysis: a proof-of-concept study
Source: BMC Med Res Methodol. 2020 Oct 28;20:266. doi: 10.1186/s12874-020-01128-2 (PMC7592566; doi:10.1186/s12874-020-01128-2)

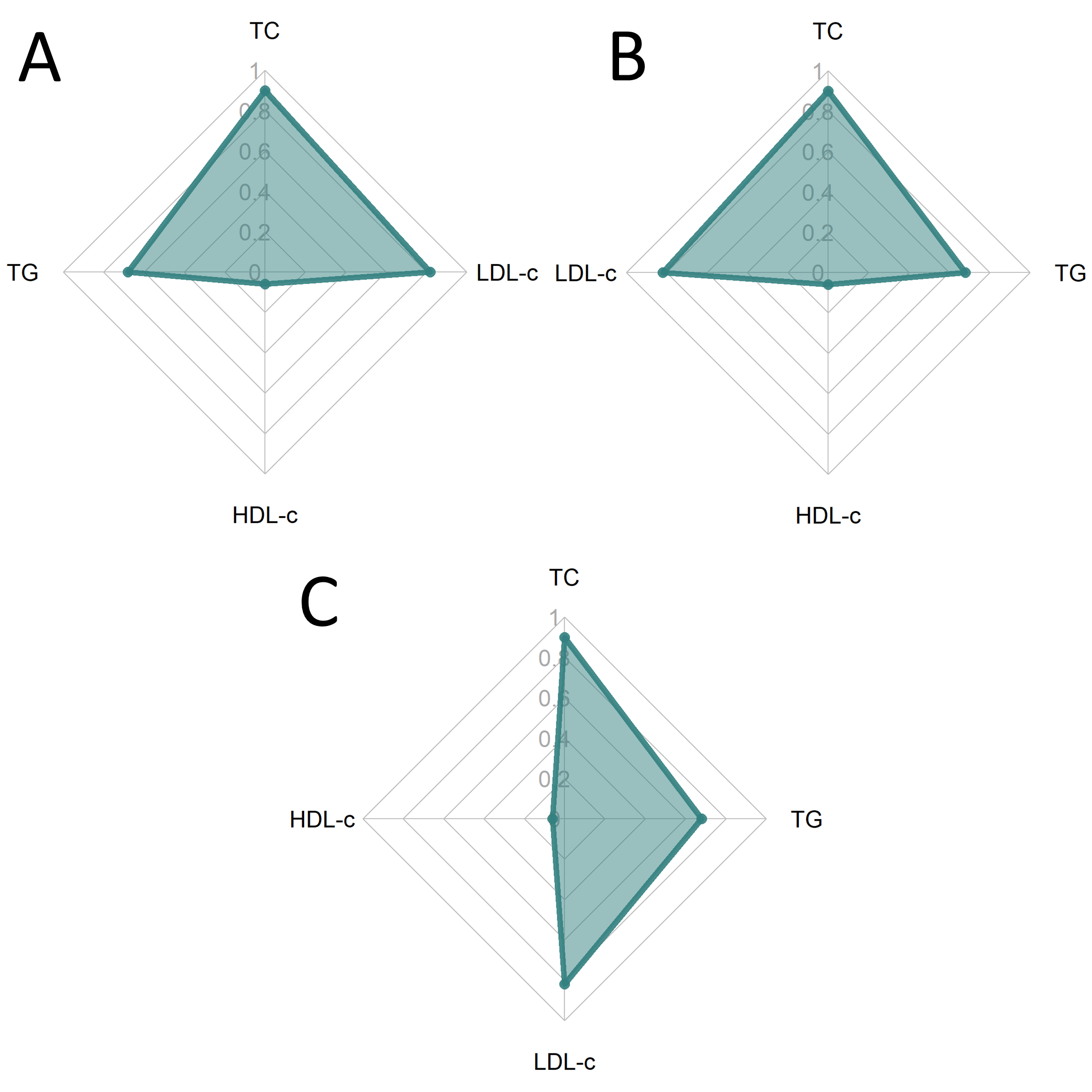

Supplement: Supplementary file 3 — Additional file 3: Supplementary Figure 2. Three possible radar plots of the SUCRA values corresponding to Safflower oil in [26]. The plots in panel A and B have the same area, since they are the same shape flipped at the vertical axes. The plot in panel C has a different area due to the different triangles formed by TC & HDL-c and TC & LDL-c. [file 12874_2020_1128_MOESM3_ESM.tif]
